# Feasibility of pressurized intra peritoneal aerosol chemotherapy using an ultrasound aerosol generator (usPIPAC)

**DOI:** 10.1007/s00464-022-09525-y

**Published:** 2022-08-29

**Authors:** Phil Höltzcke, Iaroslav Sautkin, Samuel Clere, Arianna Castagna, Alfred Königsrainer, Peter P. Pott, Marc A. Reymond

**Affiliations:** 1 Department of General and Transplant Surgery, University Hospital Tübingen, Tübingen, Germany; 2 Sinaptec SaRL, Lézennes, France; 3 National Center for Pleura and Peritoneum (NCPP), National Tumor Center SW Germany, Tübingen, Germany; 4 Institute for Medical Device Technology, University of Stuttgart, Stuttgart, Germany; 5 National, Cemter for Pleura and Peritoneum, University Hospital Tübingen, Hoppe-Seyle Str. 3, 72076 Tübingen, Germany

**Keywords:** Peritoneal metastasis, Intraperitoneal chemotherapy, Ultrasound, Drug delivery, Cisplatin, Doxorubicin, Pressurized intraperitoneal aerosol chemotherapy—PIPAC

## Abstract

**Background:**

We tested the feasibility of ultrasound technology for generating pressurized intraperitoneal aerosol chemotherapy (usPIPAC) and compared its performance vs. comparator (PIPAC).

**Material and methods:**

A piezoelectric ultrasound aerosolizer (NextGen, Sinaptec) was compared with the available technology (Capnopen, Capnomed). Granulometry was measured for water, Glc 5%, and silicone oil using laser diffraction spectrometry. Two- and three-dimensional (2D and 3D) spraying patterns were determined with methylene blue. Tissue penetration of doxorubicin (DOX) was measured by fluorescence microscopy in the enhanced inverted Bovine Urinary Bladder model (eIBUB). Tissue DOX concentration was measured by high-performance liquid chromatography (HPLC).

**Results:**

The droplets median aerodynamic diameter was (usPIPAC vs. PIPAC): H_2_0: 40.4 (CI 10–90%: 19.0–102.3) vs. 34.8 (22.8–52.7) µm; Glc 5%: 52.8 (22.2–132.1) vs. 39.0 (23.7–65.2) µm; Silicone oil: 178.7 (55.7–501.8) vs. 43.0 (20.2–78.5) µm. 2D and 3D blue ink distribution pattern of usPIPAC was largely equivalent with PIPAC, as was DOX tissue concentration (usPIPAC: 0.65 (CI 5-95%: 0.44–0.86) vs. PIPAC: 0.88 (0.59–1.17) ng/ml, *p* = 0.29). DOX tissue penetration with usPIPAC was inferior to PIPAC: usPIPAC: 60.1 (CI 5.95%: 58.8–61.5) µm vs. PIPAC: 1172 (1157–1198) µm, *p* < 0.001). The homogeneity of spatial distribution (top, middle and bottom of the eIBUB) was comparable between modalities.

**Discussion:**

usPIPAC is feasible, but its performance as a drug delivery system remains currently inferior to PIPAC, in particular for lipophilic solutions.

Interventional oncology is a rapidly growing area of modern oncology and complements multimodal therapy concepts [1]. An example is intraperitoneal (IP) chemotherapy for peritoneal metastasis (PM) [2].

The rationale for IP drug delivery is based on the pharmacokinetic advantage resulting from the peritoneal-plasma barrier potential to treat small, poorly vascularized PM adequately. The efficacy of IP chemotherapy for PM depends on various factors [3], including the mode of drug delivery [4, 5]. The known limitations of IP chemotherapy are poor drug tissue penetration and inhomogeneous spatial drug distribution [6]. An innovative drug delivery technique is Pressurized IntraPeritoneal Aerosol Chemotherapy (PIPAC) [7], which distributes the chemotherapeutic substances in the form of an aerosol. PIPAC’s rationale is manifold: using an aerosol rather than a liquid solution, PIPAC improves the homogeneity of spatial drug distribution; by applying artificial hydrostatic pressure to the abdomen, PIPAC enhances drug penetration into the tumoral tissue [8]; by compressing portal and parietal veins, PIPAC reduces blood outflow during drug application [9, 10]. Repeated PIPAC cycles are possible, which is a precondition for effective palliative chemotherapy [11]. Finally, comparing tumor biopsies between PIPAC cycles allows histological assessment of tumor response [12, 13].

Although the homogeneity of spatial distribution after PIPAC is improved compared to IP chemotherapy with liquids, this distribution remains suboptimal [14–16]. Thus, further technological development is needed to exploit PIPAC’s full therapeutic potential. A possible opportunity is to use aerosol ultrasound generators, a standard in pulmonary medicine [17]. This study evaluated the potential use of ultrasound technology to improve PIPAC’s performance as a drug delivery system. For this purpose, we tested the feasibility of PIPAC using an ultrasound generator (usPIPAC) in-vitro and ex-vivo and compared usPIPAC performance with the available CE-certified comparator.

## Materials and methods

### Study design

This in-vitro and ex-vivo study compared the performance of two devices for aerosolizing chemotherapy: (a) a test group with an 80 kHz ultrasound generator (Sinaptec SARL, Lezennes, France); and (b) a control group with a CE-certified nebulizer used in clinical routine for PIPAC (CapnoPen®, Capnopharm, Tübingen, Germany). Four parameters were compared:

- droplet size (granulometry) after aerosolization of distilled water, glucose solution, and silicone oil,
- tissue concentration of DOX,
- depth of tissue penetration (DOX),
- homogeneity of the spatial distribution of methylene blue using 2D and 3D targets.

The ideal specifications would be a smaller droplet size, a superior tissue concentration, a superior depth of tissue penetration and a more homogeneous distribution of the tracer, as compared to the available comparator [12]. All experiments were performed in triplicate; blinding was applied whenever possible.

### Sample size

This is an exploratory study. Whereas pilot data were available for the control group (PIPAC), we had no pilot data for the test group (usPIPAC). We based our hypothesis on an identical drug concentration in the tissue after usPIPAC vs PIPAC, and calculated the sample size using the T statistic and non-centrality parameter. Considering a difference ≥ 25% to be clinically significant, an α-error of 0.05 (two-tailed), a power of 0.8, and 30% standard deviation, we need a minimum of two groups of 24 biopsies, totalizing 48 biopsies. This number was reached with 2 × 3 ex-vivo models with 9 biopsies/model, in total 54 biopsies.

### Ethical and regulatory background

Since no live animals were used or sacrificed for these experiments, no authorization of the Animal Protection Committee was required. No human-derived specimens were used so that, according to the German law, no approval of the Institutional Review Board was needed.

### Technology

#### PIPAC

The aerosolizer currently used in clinical practice for PIPAC (Capnopen®, Capnomed, Zimmern o.R., Germany) uses a nozzle-based, pressure-driven miniaturized injector for aerosolizing therapeutic solutions, with a flow between 0.5 and 1.0 ml/s, a droplet velocity of 16 m/s and a driving pressure between 11 and 20 mmHg. The aerosolizer can handle an extensive range of substances, does not require continuous gas flow, and generates droplets with a bimodal size distribution (around three and 37 µm) [18]. The aerosolizer has been shown to be safe and reliable in clinical practice [19] and is CE-certified.

#### Ultrasound PIPAC (usPIPAC)

The principle of usPIPAC is to aerosolize a thin liquid phase layered at the tip of the nozzle by vibration. The ultrasound transducer used in this study (nozzle 80 kHz®, Sinaptec, Lezennes, France) uses the piezoelectric effect to convert electrical energy into mechanical movement. The transducer consists of several components:

- a titanium pavilion (“horn”): the active part of the device,
- two stacked rings of piezo-composite material: enabling generation of two polarities,
- a countermass: a heavier titanium component squeezing the piezoelectric rings, leading the ultrasounds waves to the pavilion.

Adaptation layers enable optimized energy transfer depending on the propagation medium (in this study: distilled water, glucose solution, and silicone oil).

**Fig. 1:**
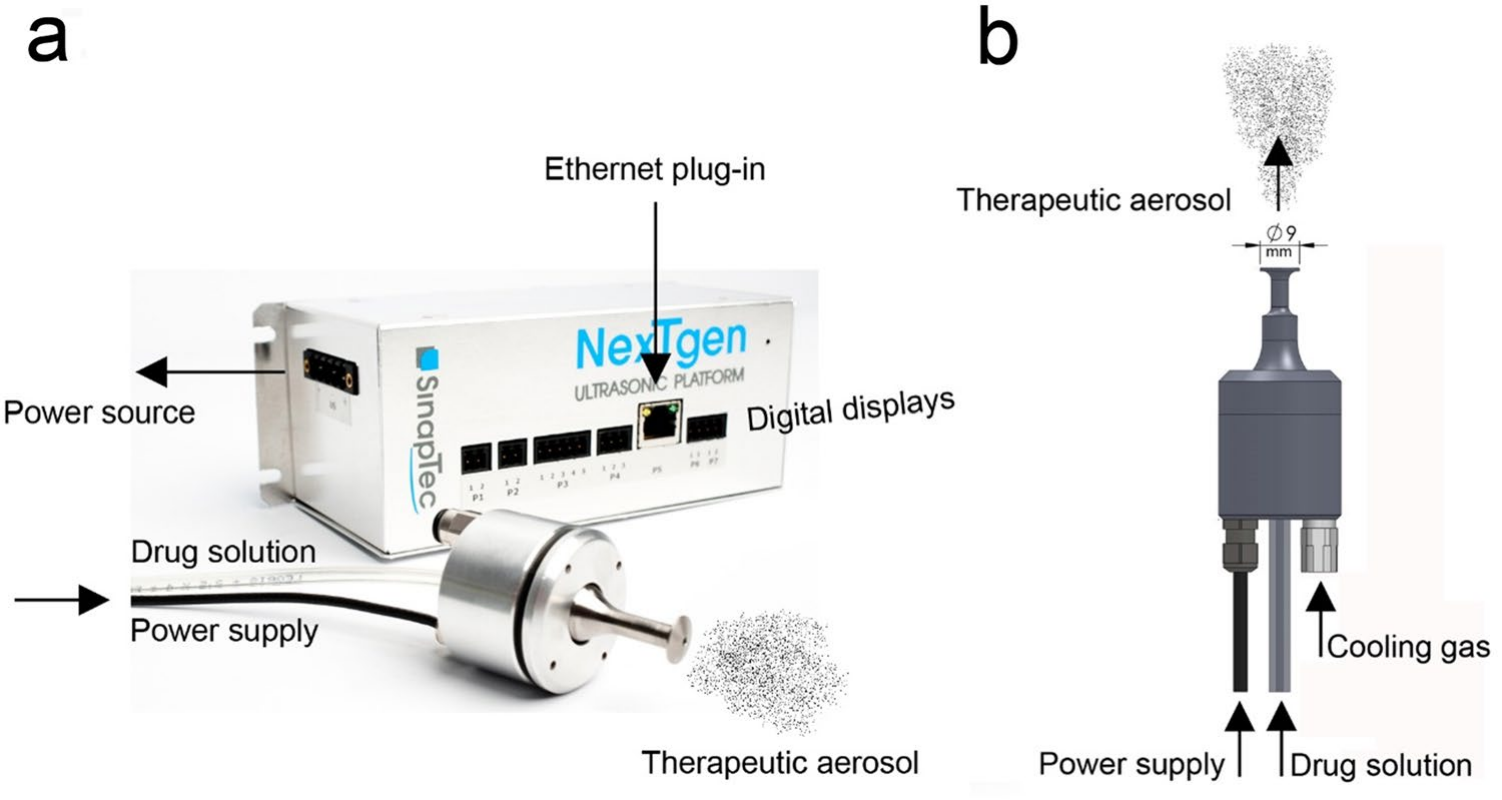
Ultrasound PIPAC (usPIPAC) technology. **a** “Inside 30” driver device and software (NexTgen ultrasonic platform); The power source (220 V) on the backside of the “Inside 30” device is not visible. **b** technical drawing of the 80 kHz piezoelectric nebulizer

A generator (Inside 30®, Sinaptec, Lezennes, France) supplies the transducer with energy, converting the network voltage (240 V) into a suitable voltage (around 150 V) and converting the 50–60 Hz network signal to a high frequency signal (80 kHz). The transducer’s optimal power is delivered at its resonance (or antiresonance) frequency (around 80 kHz). Since this frequency depends on the nature of the fluid aerosolized (in particular its viscosity) and the environment (temperature, saturation), the transducer needs to be controlled in real-time by a driver. This digital driver’s software (NexTgen®, Sinaptec, Lezennes, France) can be remote-controlled over Ethernet [20] (Fig. 1).

### Model

For this feasibility experiment, we used an ex-vivo model to respect the ARRIVE guidelines [21] of Replacement, Refinement, and Reduction for animal experiments.

#### Enhanced inverted bovine urinary bladder (eIBUB) ex-vivo model

Anatomically, bovine bladders are intraperitoneal and thus covered with parietal peritoneum. Hence, after inverting them (outside-in), their inner surface is lined with homogeneous peritoneum. Since the volume of the bovine bladder is similar to the human abdomen, this model meets the expectations for realistic ex-vivo experiments aiming to optimize intraperitoneal drug delivery [22, 23].

**Fig. 2:**
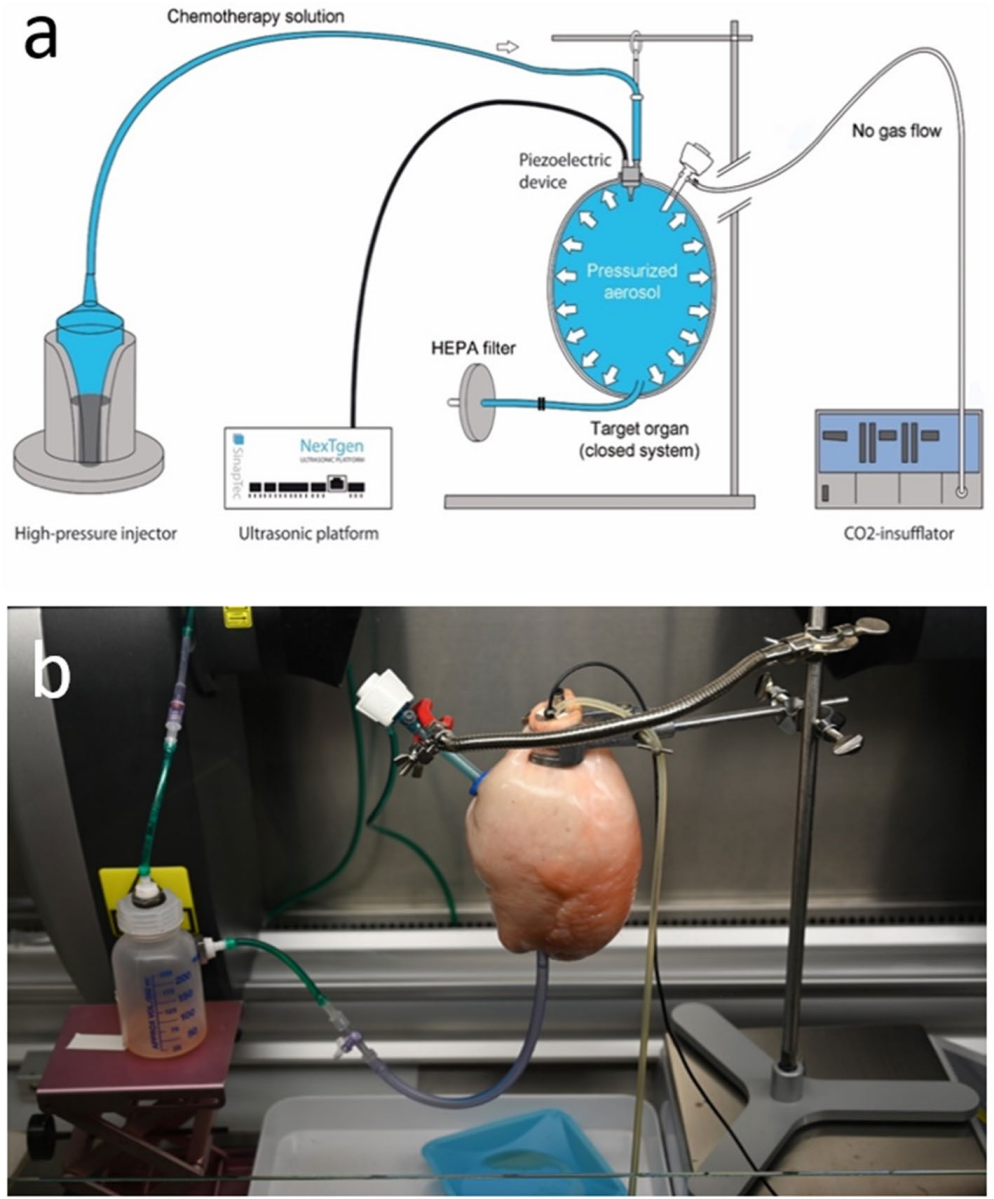
Experimental setup. The upper panel **a** shows a schema of the experimental system, consisting of the CO_2_ insufflator; the ultrasound generator (80 kHz) connected to “Inside 30” driver device (NexTgen ultrasonic platform) (Sinaptec, Lezennes, France); a high-pressure injector (AccutronHP-Thera™, Medtron AG, Saarbrücken, Germany); the organ to be treated with the therapeutic aerosol (in this ex-vivo experiment, an inverted bovine urinary bladder), the lower panel **b** shows the experimental system in reality

Fresh bovine urinary bladders were obtained from the slaughterhouse and immediately transferred to the laboratory. As shown in Fig. 2, the experimental setup consisted of the inverted bladder, a CO_2_ insufflator, the chemotherapy solution (placed in a high-pressure injector), the ultrasound device with the corresponding driver, and a filtering system for safe exsufflation.

After surgical preparation and cleaning, the bladders were inverted, and a trocar (Kii®; Applied Medial, Düsseldorf, Germany) was inserted through the bladder wall. A pneumoperitoneum was established within the bladder using an industry-standard CO_2_ insufflator (Thermoflator®, Karl Storz, Tuttlingen, Germany). The intraluminal pressure was 12–15 mmHg at room temperature (20.3 °C) with a relative air humidity of 36%. Then, 2.7 mg DOX in 50 ml NaCl 0.9% were aerosolized into the eIBUB. After 30 min exposition, the therapeutic aerosol was released into HEPA filters, and the eIBUB opened for taking biopsies.

#### Probes sampling

Nine peritoneal punch biopsies with a diameter of 8 mm were taken according to an established protocol [24] at three levels of the eIBUB (top, middle, and bottom). The surface of the probes was dried with absorbing paper, and the biopsies were placed on colored paper to guarantee proper orientation for later measurements. Afterwards, the probes were immediately deep-frozen at − 80 °C.

### Measurement methods

#### Granulometry

Granulometric measurements of aerosol particles were performed by laser diffraction spectrometry (Spraytec®; Malvern, Herrenberg, Germany). Three solutions with different viscosities were tested:

- distilled water (H_2_O): density ρH_2_O (25 °C) = 997 kg/m^3^, dynamic viscosity ηH_2_O
  (25 °C) = 1.0 mPa*s, surface tension γH_2_O(25 °C) = 71.7 mN/m,
- glucose 5% (Glc 5%, Fresenius-Kabi, Germany): density ρGlc 5%
  (17.5 °C) = 1019 kg/m^3^, dynamic viscosity ηGlc 5% (25°C) = 1.02 mPA*s, surface tension γGlc 5% near to distilled water,
- silicone oil (Elbesil- Oil B 10, Böwing GmbH, Germany); density ρE10
  (25 °C) = 945 kg/m^3^, dynamic viscosity ηE10 (25 °C) = 11.1 mPa*s, surface tension γE10 (25 °C) = 20.2 mN/m.

#### Spray pattern distribution

An ideal pattern would be an homogeneous blue staining of the whole target surface (2D-experiment) or volume (3D-experiment). For evaluating the area covered by the device, 25 ml of methylene blue (Methylene blue hydrate, Sigma-Aldrich Chemie GmbH; Steinheim; Germany) was sprayed vertically (downwards) at a distance of 10 cm onto 1) a 60 × 60 cm blotting paper (2D assessment) and 2) a cone with a diameter of 43 cm and a depth of 22 cm (3D assessment). Paper was dried at room temperature (RT). Standardized pictures were taken for each blotting paper. Then the image from the 2D target was analyzing using Image-J software (https://imagej.net/), an open-source software for processing and analyzing scientific images. After converting the pictures to grey-scale images, the pixel density was measured and a 3D-model established. After determining the edges, it was possible to define three zones: center, intermediate and periphery, and to calculate the diameter of these zones.

#### Depth of drug tissue penetration

The depth of drug tissue penetration was defined as the distance from the tissue surface where nuclear fluorescence of DOX cannot be detected anymore (edge). Five μm-thick sections from 9 biopsies (3 top, 3 middle, 3 bottom) from three bladders were cut into at right angles to the surface, fixed with Cytoseal-xyl® on a glass slide, and covered. After air-drying at RT for 20 min, the depth of tissue penetration was measured by fluorescence microscopy (DMRBE; Leica Microsystems, Wetzlar, Germany) with Leica Qwin 2002 software. Nuclear fluorescence was determined at an emission wavelength of 490 nm and absorption wavelength from 560 to 590 nm. Measurements were performed in triplicate for each slide (2 × 243 measures in total) by a trained scientist (Phil Höltzcke) blinded to the sample origin.

#### Drug tissue concentration

##### Pre-analytical sample preparation

After thawing at RT, the vials were placed into a Speedvac device (S-Concentrator, BA-VC-300H; H. Saur, Laborbedarf, Reutlingen, Germany) and centrifuged under vacuum overnight (1000 rpm; 100 mbar) at RT for lyophilization. After weighting, the dry pellets were rehydrated with 1.5 ml distilled water and homogenized using a Thermomixer comfort (Eppendorf Vertrieb Deutschland GmbH; Hamburg; Germany) at 1400 rpm for 15 min at RT. Then, the probes were disrupted using a homogenizer (TissueLyser LT; QIAGEN GmbH, Hilden, Germany). Finally, the tubes were centrifuged at 11,000 rpm for 15 min at RT, and stored at − 80 °C.

##### Doxorubicin concentration measure using high-performance liquid chromatography (HPLC)

The tissue concentration measurements of DOX were performed by an external, GLP-certified laboratory (MVZ Dr. Eberhard & Partner, Dortmund, Germany). The laboratory was blinded to the origin of the samples (technology used, the position of the biopsies, etc.). The DOX concentration was measured by high-performance liquid chromatography (Waters Fluorescence Detector 2475; Waters Inc., Milford, MA, USA), with a serum lower limit of quantification (LLoQ) of 5 ng/ml. Pre-analytical validation proved a linear range of Glc 5% matrix measurements from 0.1 to 10,000 μg/ml DOX and established no interference by the organic matrices.

#### Homogeneity of spatial distribution

Homogeneity of spatial distribution in the target tissue was determined by comparing DOX tissue concentration and penetration at different locations (the top, middle, and bottom of the eIBUB) treated during the usPIPAC experiments. The measurement methods are described above. The H0-hypothesis (perfect distribution) assumed identical results between locations. In a second step, depth and concentration values were compared with those obtained with PIPAC using nozzle technology. The H0-hypothesis assumed identical results of usPIPAC vs. available comparator (PIPAC).

### Occupational health safety

The ex-vivo experiments performed in this study involved DOX (Doxorubicin HCl Teva ®, Teva, Ulm, Germany), presenting a potential occupational health hazard. All experiments were performed by qualified personal in the NCPP laboratory, which was certified for manipulating toxic aerosols in fall 2016. All spraying experiments were performed in a class-3 safety hood and remote-controlled.

### Statistical analysis

Descriptive statistics: Continuous data are expressed as the mean and confidence intervals 5–95% or, when meaningful, as median values. Comparative statistics: Means between groups were compared by the Mann–Whitney U test or repeated variance analysis (ANOVA) with the help of SPSS software 25 (IBM Corp., Armonk, NY, USA).

## Results

The aerosol characteristics obtained with the available comparator based on nozzle technology indicated a mean aerodynamic droplet size around 37 µm, the ability to aerosolize solutions with various viscosities but a suboptimal homogeneity of the spatial distribution. In this study, we tested the feasibility of usPIPAC and compared its performance with the CE-certified device currently used for PIPAC in patients.

### Feasibility

usPIPAC was feasible on the IBUB model ex-vivo. Importantly, no gas flow was needed for aerosolizing the therapeutic solution. No cooling gas was needed either. Notably, the transducer (with a diameter of 28 mm), and not only the trump (diameter 9 mm), needed to be inserted into the bladder wall since the trump is oscillating, generating local heat and possibly microscopic tissue damage. The transducer was not sterile and was re-used several times after intermediate cleaning with propanol solution.

### Granulometry

**Fig. 3:**
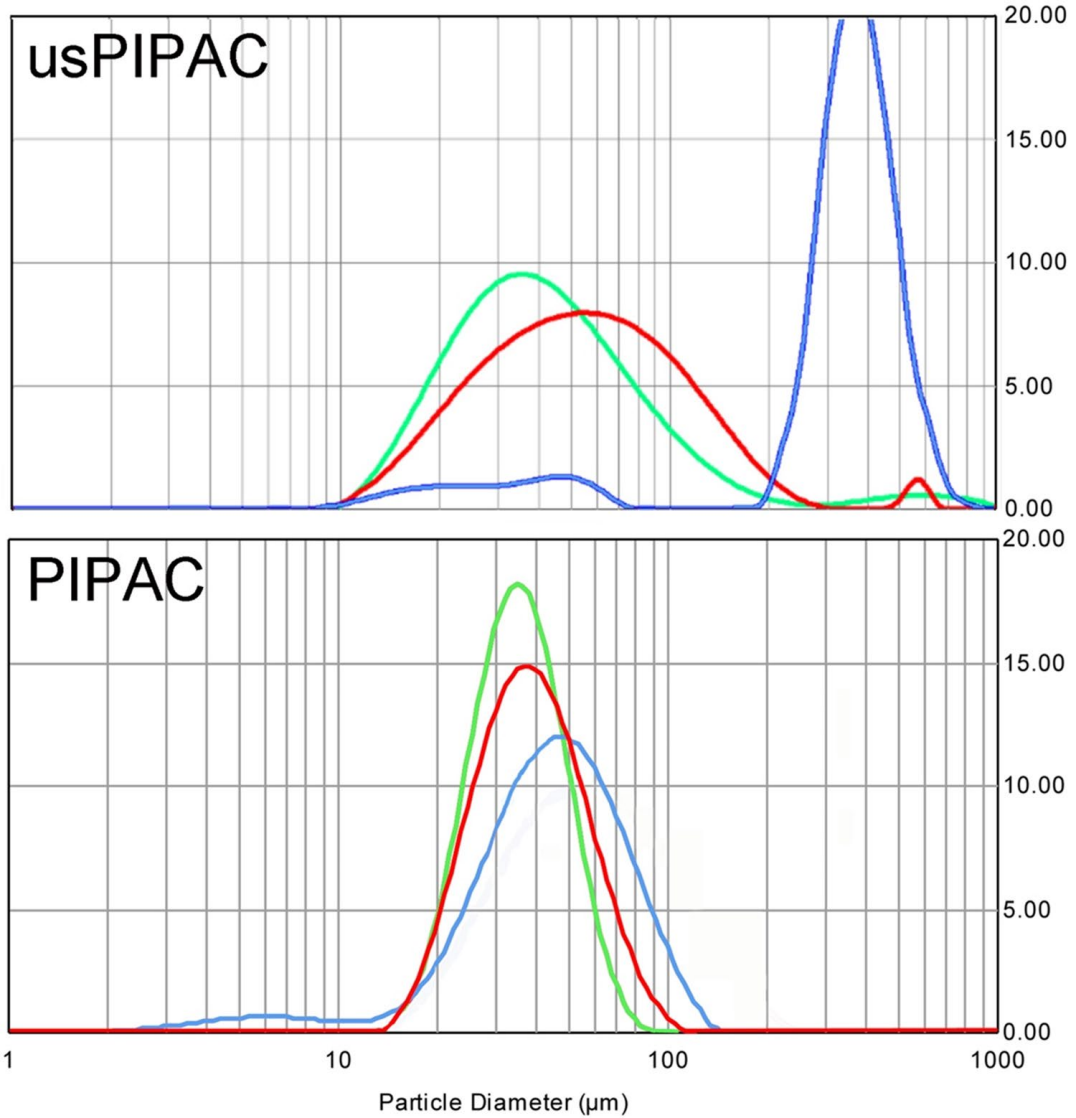
Granulometry of usPIPAC vs. PIPAC. Frequency distribution of median aerodynamic droplet size for distilled water (= green), Glc 5% (= red), and silicone oil (= blue) for usPIPAC (upper panel) and PIPAC (lower panel). *X*-axis: median aerodynamic droplet size (µm); *Y*-axis: frequency (colour figure online)

In our study, the aerosol granulometry was determined by laser diffraction spectrometry in a dry environment at RT. As illustrated in Fig. 3, the median aerodynamic diameter (MAD) of the usPIPAC aerosol droplets was broadly comparable with the comparator when water (40.4 µm, CI 5-95%: 19.0–102.3) or aqueous solutions (52.8 µm, CI 5-95%: 22.2–132.1) were aerosolized, but increased by two orders of magnitude with silicone oil (178.7 µm, CI 5-95%: 55.7–501.8).

### Spray pattern distribution

**Fig. 4:**
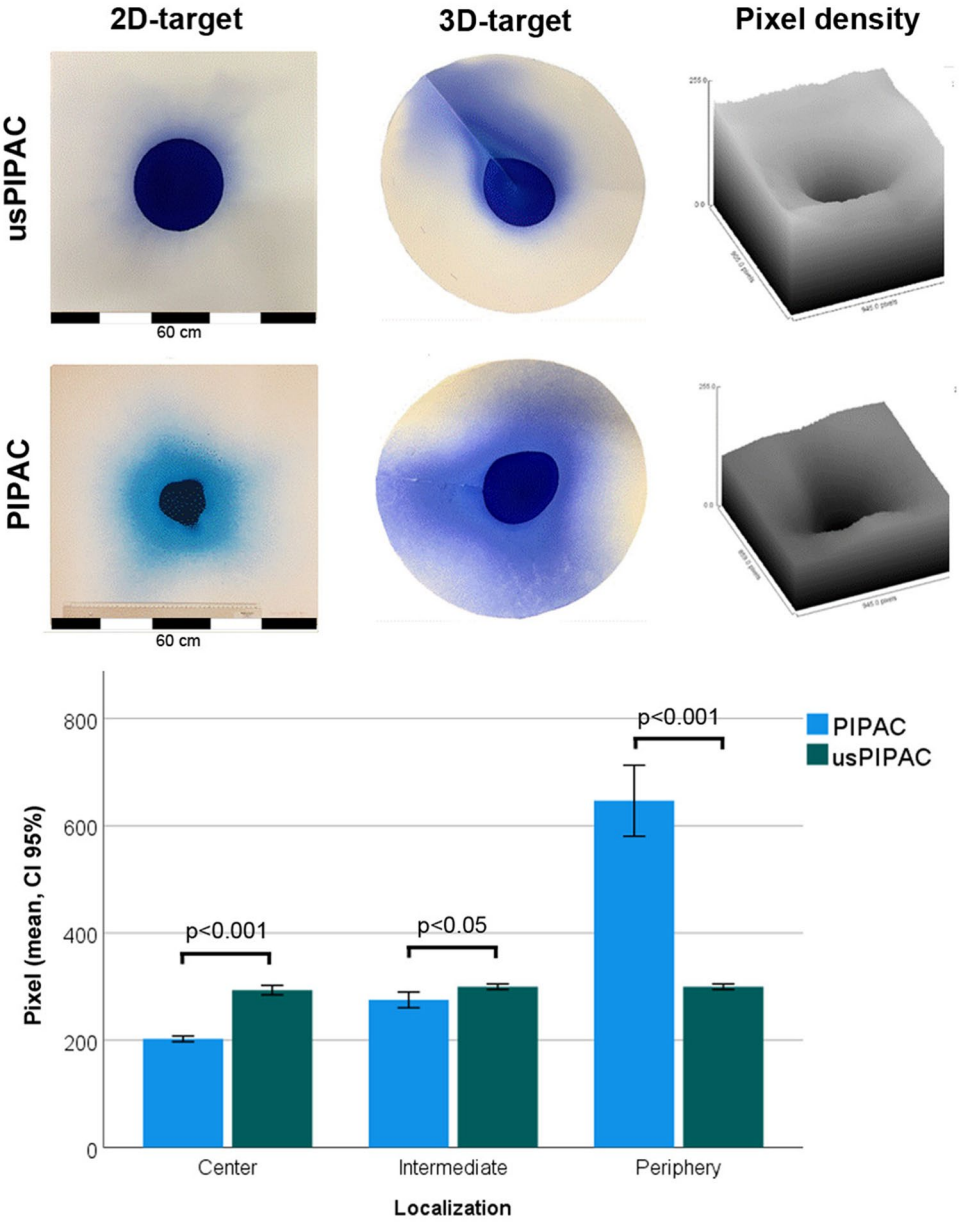
Spatial distribution of blue ink aerosolized with usPIPAC vs. PIPAC. The 2D and 3D blue ink distribution pattern of usPIPAC was largely equivalent with PIPAC (better in the center, inferior in the periphery) The upper pictures show the respective staining pattern on a flat surface (2D target) and a cone (3D-target). The 2D-picture is then transformed by image analysis (using Image-J software) into a 3D- model (*x* and *z*-axis: pixel position on the target; *y*-axis; pixel intensity). Then the pixel intensity is compared at the center, in the intermediate zone, and in the periphery of the target. Dimensions of the blotting paper: 60 × 60 cm. Cone diameter: 43 cm, depth 22 cm. Spraying distance:10 cm

Figure 4 shows the spray distribution of 30 ml blue ink with usPIPAC vs. PIPAC on a blotting paper. Spatial distribution is not homogeneous for both modalities. Two zones can be characterized: an inner zone with intense staining, corresponding to the impaction zone of the aerosol directly in front of the device; an outer area with lighter staining, corresponding to the deposition zone of floating, tiny aerosol droplets.

When blotted on a 2D target, the inner zone is more extensive for usPIPAC than for PIPAC, which is a favorable property. However, this difference is not observed on a 3D target. The surface coverage of the 3D target is broader after PIPAC vs. usPIPAC. Preferred directional staining to the left upper zone is observed after usPIPAC.

### Depth of drug tissue penetration

Depth of tissue penetration was measured by fluorescence microscopy, determining nuclear staining with DOX. Considerable differences were observed between (60.1, CI 5-95%: 58.8–61.5 µm) vs. PIPAC (1172, CI 5-95%: 1157–1198 µm), *p* < 0.001 (Kruskal–Wallis).

**Fig. 5:**
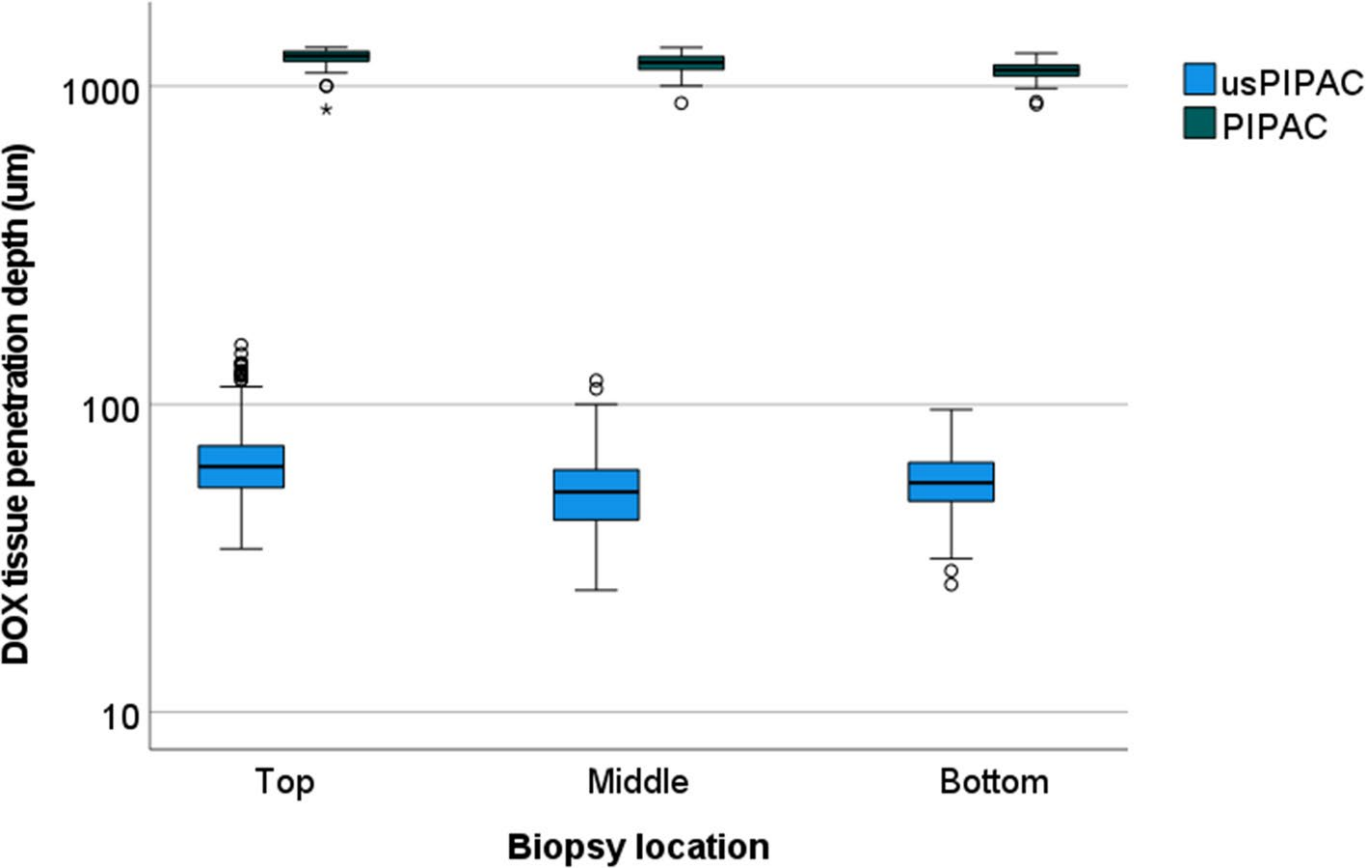
Depth of DOX tissue penetration after usPIPAC vs. PIPAC. Boxplot depth of DOX tissue penetration usPIPAC (blue) vs. PIPAC (green) at three biopsy locations (top, middle, and bottom of the eIBUB model). Logarithmic scale. Depth of DOX tissue penetration after PIPAC is superior to usPIPAC, by one order of magnitude (Kruskal–Wallis, *p* < 0.001) (colour figure online)

**Fig. 6:**
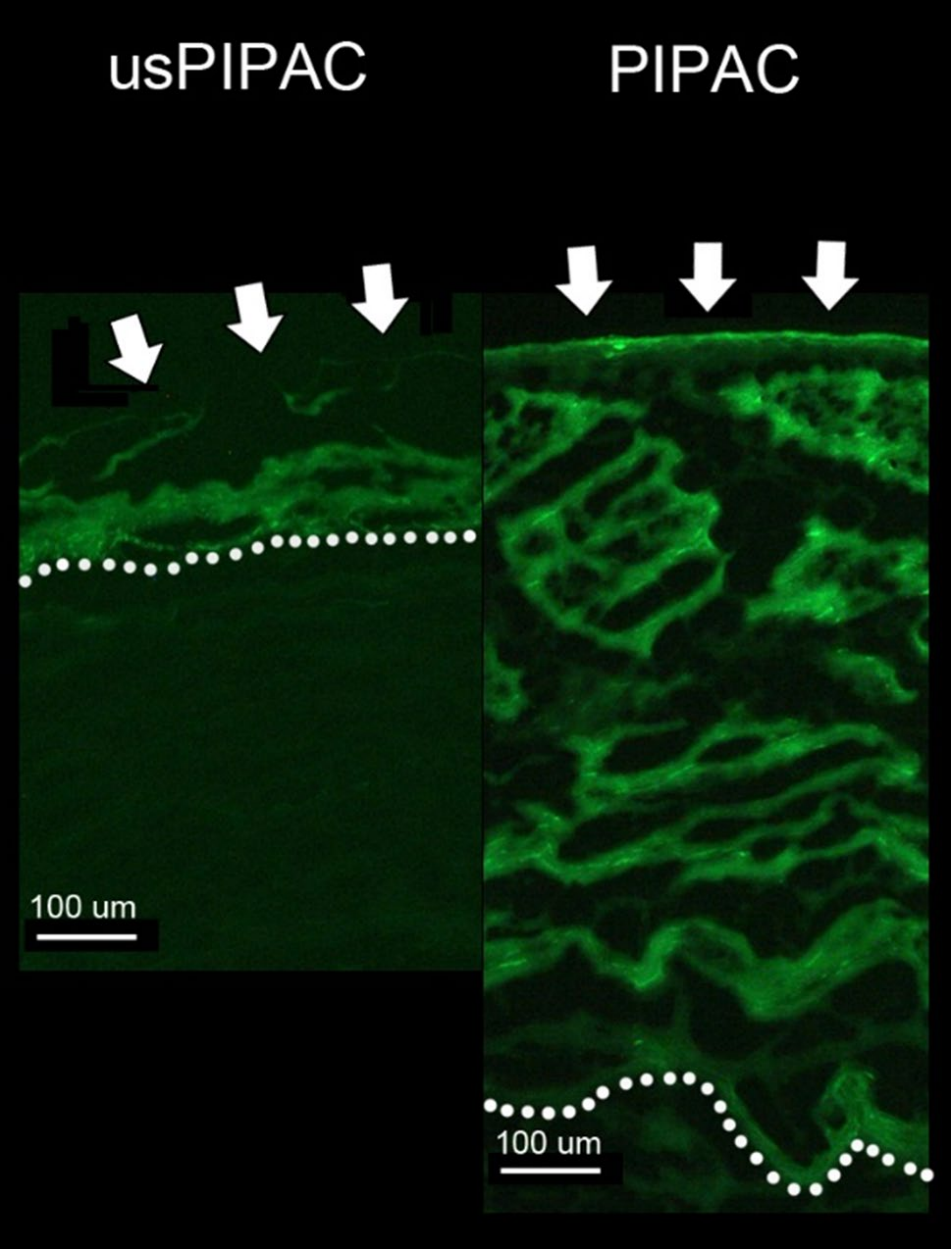
Tissue of immunofluorescence of DOX after usPIPAC vs. PIPAC. Representative fluorescence microscopy pictures of nuclear staining with DOX. The drug penetrates the tissue deeper after PIPAC vs. usPIPAC. Magnification ×10

Figure 5 illustrates these differences: the median depth of DOX tissue penetration after PIPAC (green boxplots) is superior to 1 mm, whereas tissue penetration does not exceed 0.1 mm after usPIPAC (blue boxplots). Figure 6 shows a representative example of tissue immunofluorescence of DOX, aerosolized as usPIPAC (left panel) vs. PIPAC (right panel).

### Drug concentration in tissue

**Fig. 7:**
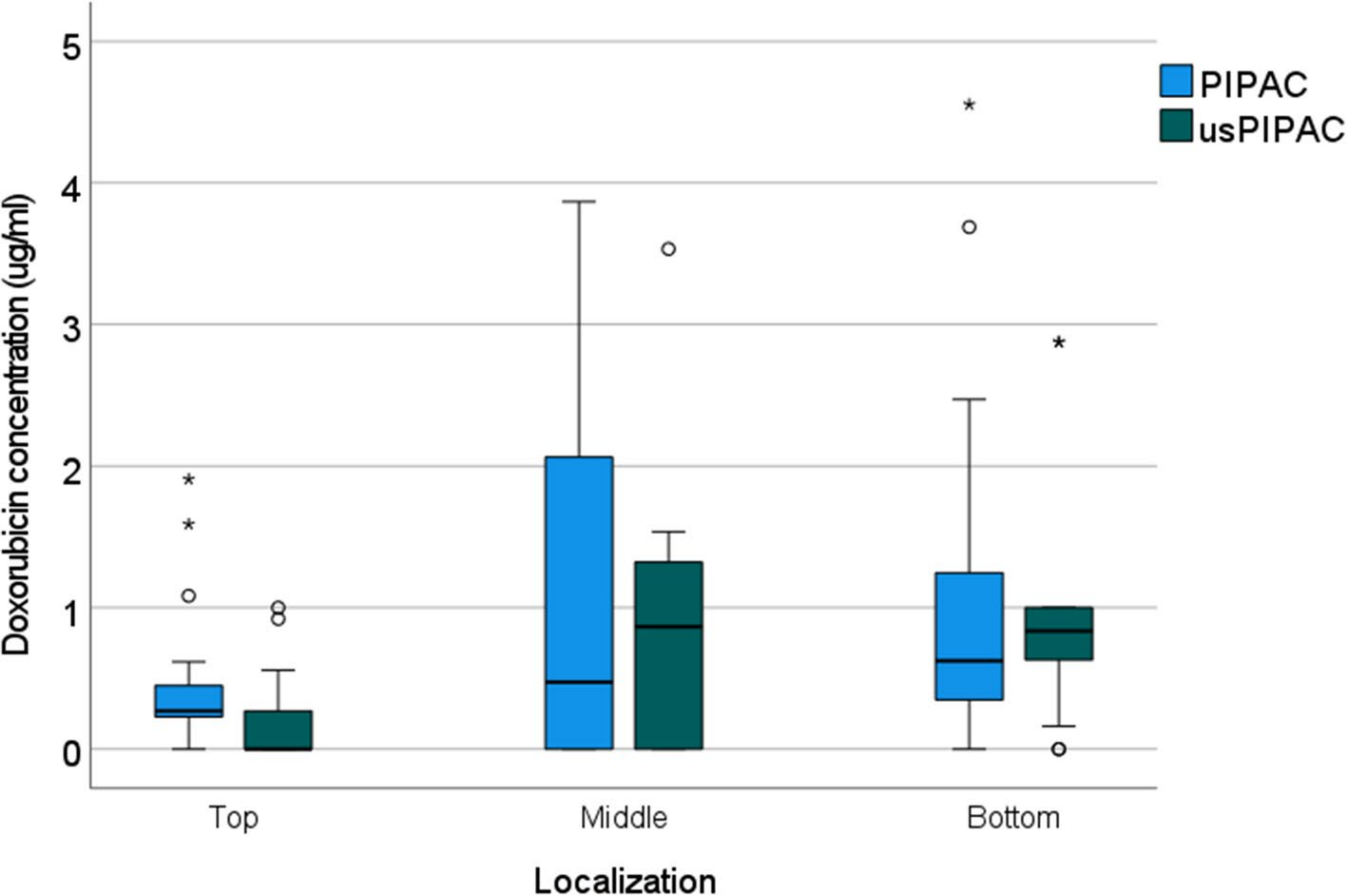
DOX tissue concentration after usPIPAC vs. PIPAC. Depth of DOX tissue concentration after usPIPAC (green) vs. PIPAC (blue) at three biopsy locations (top, middle, and bottom of the eIBUB model). DOX tissue concentration after PIPAC is largely equivalent to usPIPAC (Kruskal–Wallis, *p* = 0.29) (colour figure online)

DOX concentration in peritoneal biopsies was measured by HPLC at different locations. From Fig. 7 it is apparent that DOX tissue concentration after usPIPAC (0.65, CI 5-95%: 0.44–0.86 ng/ml) was slightly inferior to PIPAC (0.88, 0.59–1.17 ng/ml, *p* = 0.29) but this difference did not reach statistical significance (Kruskal–Wallis, *p* = 0.29).

### Homogeneity of spatial distribution

Homogeneity of spatial distribution was measured by comparing the depth of tissue penetration and tissue concentration of DOX at different locations (top, middle, or bottom). As shown in Fig. 5, the depth of tissue penetration did not depend on the biopsy position within the bladder, suggesting a homogeneous spatial distribution. However, an increasing gradient of DOX tissue concentration was observed from the top to the bottom of the target organ, as illustrated in Fig. 7 (Kruskal–Wallis, *p* < 0.001).

## Discussion

PIPAC’s aim is to optimize drug delivery into the tumor nodes, with the goal of achieving a cytotoxic drug concentration in the whole target tissue. However, an aerosol is not a gas and tissue uptake is caused by impaction and gravitation forces. Furthermore, recent studies have shown that there are significant differences in drug concentration in the peritoneal tissue, depending on the anatomic localization [25], the aerosolizer used [14, 26], the position of the nozzle [27], differences in tissue nature [28], and preanalytical biopsy handling [24].

Future aerosolizers are expected to improve homogeneity of drug distribution in the peritoneal tissue. A possible improvement is to use ultrasound technology for generating therapeutic aerosols, for example with piezo-electric devices. Such a technology has already been used in the very first PIPAC prototype developed in 1999 [29]. However, using ultrasound devices with continuous gas flow is challenging: continuous gas inflow into the abdomen might increase the intraabdominal pressure so that a continuous outflow is required. Under such conditions, there is a preferential distribution of the aerosol droplets from the inflow to the outflow. It is challenging to determine the actual dose reaching the target tissue, which can only be a fraction of the total dose applied. Finally, a continuous gas flow in an open system raises significant concerns for occupational health safety, since it is impossible to verify the tightness and exclude environmental contamination.

In this study, we show for the first time the feasibility of PIPAC using an ultrasound generator (usPIPAC). The ultrasound transducer used in this study uses the piezoelectric effect to convert electrical energy into mechanical movement. A thin liquid phase layered at the tip of the nozzle is aerosolized by vibration. No gas flow is needed. Notably, the technology used does not rely on the principle of hydrodynamic cavitation [30].

For aqueous solutions, usPIPAC was able to generate an aerosol with a droplet diameter largely comparable to current PIPAC technology. However, the droplet size increased dramatically when oil was aerosolized. This is indeed a handicap since homogeneity of spatial distribution is inversely proportional to the droplet size. Hence, usPIPAC is not well suited for aerosolizing lipophilic solutions with a higher viscosity. Similarly but to a lesser degree, the ultrasound device used in this study was less suitable than the nozzle-based technology for aerosolizing glucose solutions.

The 2D and 3D blue ink distribution pattern of usPIPAC was largely equivalent with PIPAC. Specifically, when blotted on a 2D target, the inner stained zone was more extensive for usPIPAC than PIPAC, which is a favorable property. However, this difference was not observed on a 3D target, suggesting a different geometry of the spraying cone. Moreover, peripheral staining was superior after PIPAC vs. usPIPAC. Preferred directional staining between 270° and 360° was observed after usPIPAC, suggesting external influence (possibly airflow generated by the room ventilation system) on the aerosol-cloud deposition.

The drug concentration and penetration in the target peritoneal tissue, as determined in our ex-vivo inverted bovine urinary bladder model, showed inferiority of usPIPAC vs. conventional PIPAC. This inferiority was highly significant for the depth of drug penetration into the tissue, which differed by an order of magnitude (60 vs. 1172 µm). Drug concentration in the target tissue was also less after usPIPAC than PIPAC (0.65 vs. 0.88 ng/ml) but the difference did not reach statistical significance.

The present research fits well into the current PIPAC research map, at a timepoint when multiple technologies are developed to further improve the clinical efficacy of this promising drug delivery system. The usPIPAC technology used in this study has several advantages: No gas flow is needed during the application, the small size of the trump (9 mm diameter) allows minimally invasive use, the flow of 0.1 ml/s allows aerosolization of larger volumes of therapeutic solutions, the technology can aerosolize aqueous or oily substances, and the device can be controlled remotely. However, the pharmacokinetics results obtained with usPIPAC in this ex-vivo study are, at this stage of development, rather disappointing. Anyhow, at this stage, we would not exclude that usPIPAC might become a leading technology for intraperitoneal drug delivery in the future. Further preclinical and clinical comparative studies are needed to determine which aerosolizing technology is best suitable for PIPAC, including 2nd-generation nozzle devices [27], usPIPAC, electrostatic precipitation (ePIPAC) [31], and hyperthermia (hPIPAC) [32].

## Abbreviations

(us)PIPAC: (Ultrasound) pressurized intraperitoneal aerosol chemotherapy
2D/3D: Two-/three-dimensional
DOX: Doxorubicin
eIBUB: Enhanced inverted bovine urinary bladder
HPLC: High-performance liquid chromatography
IP: Intraperitoneal
PM: Peritoneal metastasis
ARRIVE: Animal research: reporting of in-vivo experiments
HEPA: High efficient particulate air
RT: Room temperature
GLP: Good laboratory practice
NCPP: National center of pleura and peritoneum
ANOVA: Analysis of variance
CI: Confidence interval
